# Management of retinopathy of prematurity in a tertiary referral neonatal intensive care unit: Treatment rates and the impact of outsourcing laser therapy

**DOI:** 10.1111/aos.70029

**Published:** 2025-11-25

**Authors:** L. A. Derks, H. R. Taal, S. E. Loudon, I. K. M. Reiss, J. R. Vingerling, A. M. Tjiam

**Affiliations:** ^1^ Department of Ophthalmology Erasmus University Medical Centre Rotterdam The Netherlands; ^2^ Division of Neonatology, Department of Neonatal and Paediatric Intensive Care Erasmus University Medical Centre Rotterdam The Netherlands

**Keywords:** incidence, organisational change, panretinal photocoagulation treatment, retinopathy of prematurity, screening

## Abstract

**Purpose:**

To describe the treatment rate for retinopathy of prematurity (ROP) at a tertiary referral neonatal intensive care unit (NICU) in the south‐western region of the Netherlands. In addition, we evaluated the impact of outsourcing laser treatment, implemented in 2018, by comparing treatment characteristics from 3 years prior and 3 years following this organisational change.

**Methods:**

This retrospective observational cohort study evaluated data of preterm infants born between 2015 and 2020 who were admitted to our NICU and met the eligibility criteria for ROP screening. ROP treatment rate was calculated for infants screened in our NICU. The impact of outsourcing laser treatment was evaluated based on ROP type at treatment decision, time between treatment decision and treatment, and adverse outcomes, including additional treatments and retinal detachment.

**Results:**

A total of 358 infants were screened at our NICU between 2015 and 2020; complete data were available for 343 infants, of whom 15% (51/343) required treatment. Since outsourcing treatment, additional treatment secondary to laser was performed less frequently (25%, 5/20 versus 7%, 2/31). However, the mean number of days between treatment decision and treatment increased since outsourcing treatment (*p* = 0.014), and 32% (9/28) of cases were treated prior to reaching the criteria of type 1 ROP.

**Conclusion:**

The treatment rate among infants screened at our tertiary referral NICU exceeds previous national and hospital‐level reports, likely due to early transfer of low‐risk cases. Outsourcing ROP treatment might have led to improved primary treatment success; however, possible disadvantages include treatment delays and over‐treatment.

## 1 INTRODUCTION

Retinopathy of prematurity (ROP) is characterised by abnormal development of the retinal vessels in preterm infants and can potentially lead to visual impairment or blindness (Blencowe et al., 2013; Gilbert, 2008; Hellström et al., 2013). Routine screening and timely treatment are essential means of secondary prevention of vision loss due to ROP (Blencowe et al., 2013; Gilbert, 2008). Widespread access to ROP screening and treatment has resulted in a relatively low incidence of ROP‐related vision loss in high‐income countries such as the Netherlands (Gilbert, 2008; Gilbert et al., 2019). However, the latest evaluation of ROP screening in the Netherlands (NEDROP 2) showed that although the incidence of ROP had remained stable since 2009, the number of severe cases of ROP had increased, and subsequently, the yearly number of infants that required treatment had doubled between 2010 and 2017 (Trzcionkowska et al., 2023; Trzcionkowska, Groenendaal, et al., 2021). Nonetheless, the absolute number of ROP treatments performed in the Netherlands remains low. In 2017, 39 infants were treated for ROP, which constitutes to less than 3% of all live born infants born before 32 weeks gestational age (Perined, 2022; Trzcionkowska et al., 2023).

Low treatment rates have led to discussions regarding the need for a more centralised organisation of ROP treatment in the Netherlands. Centralisation of surgical treatment is intended to improve the overall quality of care. The rationale is that a higher caseload for a smaller, more specialised group of surgeons creates the highest level of expertise regarding specific treatment indications, treatment procedures, complications and post‐operative follow‐up. For other rare paediatric conditions that require surgical management, studies have demonstrated improved outcomes after centralisation of care (Davenport et al., 2004, 2011; Leoni et al., 2022; Söderström et al., 2024; Vonlanthen et al., 2018). In the Netherlands specifically, there have been successful efforts to centralise both paediatric oncological treatment and paediatric neonatal surgery on a national level (van der Steeg et al., 2023; Wijnen & Hulscher, 2022). Whether ROP treatment could be organised in a similar manner, and whether this would positively impact the quality of care and long‐term visual outcomes, has yet to be investigated.

Erasmus University Medical Centre (EMC) is a tertiary referral hospital with a level IV Neonatal Intensive Care Unit (NICU) (Stark et al., 2023) that cares for 650–700 patients a year. As a level IV NICU hospital, EMC provides high‐level medical care and surgery for newborns, including the smallest and sickest extremely preterm infants. Although the NEDROP studies have provided ample insight into ROP screening and treatment across NICU centres of all different levels in the Netherlands, the incidence rates of ROP and ROP treatment specifically in a high‐risk level IV NICU population are unknown. Moreover, starting from 2018, EMC was compelled to outsource laser treatment for ROP to another hospital due to a shortage of specialised staff. The effects of this change in the organisation of ROP treatment have not yet been evaluated, but could provide insights relevant to the discussion surrounding the centralisation of care.

This retrospective observational study aimed to assess the rate for ROP treatment among infants screened in a tertiary care setting over a 6‐year period, and to evaluate the impact of outsourcing laser treatment, which was introduced in 2018, by comparing treatment characteristics before and after its implementation.

## 2 METHODS

### 2.1 Study design and setting

This retrospective observational cohort study reviewed data of preterm infants admitted to the tertiary referral NICU at EMC, who were born between 1 January 2015 and 31 December 2020, and met the eligibility criteria for ROP screening. This 6‐year timeframe allows for an evaluation of ROP‐related care, divided into two 3‐year periods before and after the implemented change to the organisation of ROP treatment. The national ROP screening and treatment guidelines remained unchanged from 2015 to 2020. As part of a regionalised neonatal care system, all (preterm) infants requiring high‐level neonatal intensive care in Rotterdam and the surrounding regions are admitted to the tertiary referral NICU at EMC. Once the infants' condition stabilises and no longer necessitates intensive support, they are generally transferred to regional post‐intensive care units or nurseries (level I or II NICUs) for convalescent care closer to home, and are subsequently discharged. The organisation of neonatal care in the region remained unchanged during the study period.

### 2.2 Data source and patient selection

We queried our institutional NICU patient registry for infants born between 2015 and 2020 eligible for ROP screening in accordance with the 2013 Dutch ROP Screening Guidelines (Table S1) (Nederlands Oogheelkundig Gezelschap, 2013). According to these guidelines, infants born <30 weeks gestational age and/or with a birthweight <1250 grams are eligible for ROP screening. Infants born between 30–32 weeks gestational age and/or with a birthweight between 1250–1500 grams should be screened only in the presence of one or more predetermined ROP risk factors (mechanical ventilation, sepsis, necrotising enterocolitis, postnatal corticosteroids or treatment with inotropes). In the case the exposure to these risk factors is unknown, it is advised to screen all infants born <32 weeks gestational age and/or with a birthweight <1500 grams. First screening should take place 5 weeks after birth, but not before 31 weeks post‐menstrual age.

Infants without documented parental consent were excluded from this study. Data of included infants were extracted from electronic patient files and ROP screening forms. Post‐transfer ROP screening and/or treatment data were collected from discharge letters from the hospital the infant was transferred to.

### 2.3 Demographics

Demographic variables included gestational age (GA) at birth, birthweight (BW), sex, multiple birth, ROP diagnosis and the presence of the following ROP risk factors: artificial ventilation, treatment with nitric oxide (NO), sepsis, necrotising enterocolitis (NEC), postnatal treatment with corticosteroids, treatment with inotropes. Detailed definitions of these risk factors are provided in Table S2. In addition, a cumulative ‘risk summary score’ ranging from 0 to 5 was calculated based on the number of risk factors present for each individual infant. ROP diagnosis was classified according to the *International Classification of ROP* (ICROP 2) (International Committee for the Classification of Retinopathy of Prematurity, 2005), which categorises ROP based on disease stage, zone of maximum retinal vascularisation and the presence of plus disease. The ROP diagnosis that was used was based on the maximum ROP staging observed, which was defined as the highest ROP stage recorded until the completion of ROP screening or until death occurred. At the tertiary referral NICU, ROP screening was conducted using binocular indirect fundoscopy (BIO) or a retinal camera, the latter of which was first implemented in 2017, by various ophthalmologists.

### 2.4 Outcome measures

To assess the ROP treatment rate, we calculated the proportion of infants treated within the population of infants that received at least one ROP screening at our hospital. In addition, we conducted an exploratory analysis of all eligible NICU admissions who survived to first ROP screening, regardless of screening location, to gain insight into the need for treatment among infants transferred from the tertiary referral NICU to lower‐level care settings prior to first screening.

To assess the effect of outsourcing ROP treatment, we evaluated the following outcome measures: ROP type at the time of treatment decision, the time interval to treatment, the occurrence of additional treatments and the occurrence of retinal detachment. These outcomes were analysed based on the time period in which the infant received treatment, either before (2015–2017, i.e., Period 1) or after (2018–2020, i.e., Period 2) outsourcing was implemented. In addition, an as‐treated analysis was performed to compare outcomes between infants treated on‐site and those referred for treatment, regardless of treatment period. ROP type at treatment decision was categorised according to the *Early Treatment of Retinopathy of Prematurity* (ETROP) criteria (Good WV & Early Treatment for Retinopathy of Prematurity Cooperative Group, 2004). We defined the time interval to treatment as the number of days between the decision to treat and actual treatment taking place. Throughout the entire study period, including prior to outsourcing treatment, cases of retinal detachment (Stage 4 or 5 ROP) were discussed with a specialised vitreoretinal surgeon at the tertiary referral NICU centre to which treatment was later outsourced. This hospital will be referred to as the ‘ROP referral centre’ hereafter.

### 2.5 Statistical analysis

Numerical variables were reported as median (minimum–maximum and/or interquartile range [IQR]) or mean (minimum‐maximum and/or standard deviation [SD]). Categorical variables were reported as number (*n*) and proportion (%). Demographic variables were compared based on death or transfer prior to first ROP screening. To determine the effect of outsourcing ROP treatment, ROP type at treatment decision, time interval to treatment (days), occurrence of additional treatment and occurrence of retinal detachment were compared between Period 1 and Period 2 and between on‐site and referred treatment. In the above comparative analyses, the *p*‐values for numerical variables were calculated with the Mann–Whitney *U*‐test or independent sample *t*‐test. Where applicable, mean difference between groups was presented with a 95% confidence interval (95% CI). For categorical variables, the *p*‐values were calculated with the Pearson's chi‐squared test. For the multiple comparisons in the comparative analyses, Bonferroni correction was applied. Pairwise deletion was used to handle missing data in the analyses. Statistical analysis was performed using IBM SPSS Statistics for Windows, version 28 (IBM Corp., Armonk, NY).

### 2.6 Ethics

This study was approved by the institutional research review board at EMC. Handling of data was in compliance with the EU General Data Protection Regulation and the Dutch Act on Implementation of the General Data Protection Regulation. Because of the retrospective observational nature of this study, the Dutch Medical Research Involving Human Subjects Act did not apply. As part of standard care, parents are asked for consent to use of their child's data for future medical research when admitted to our NICU. Researchers adhered to the Declaration of Helsinki while conducting this study.

## 3 RESULTS

### 3.1 Patient selection and demographics

**FIGURE 1:**
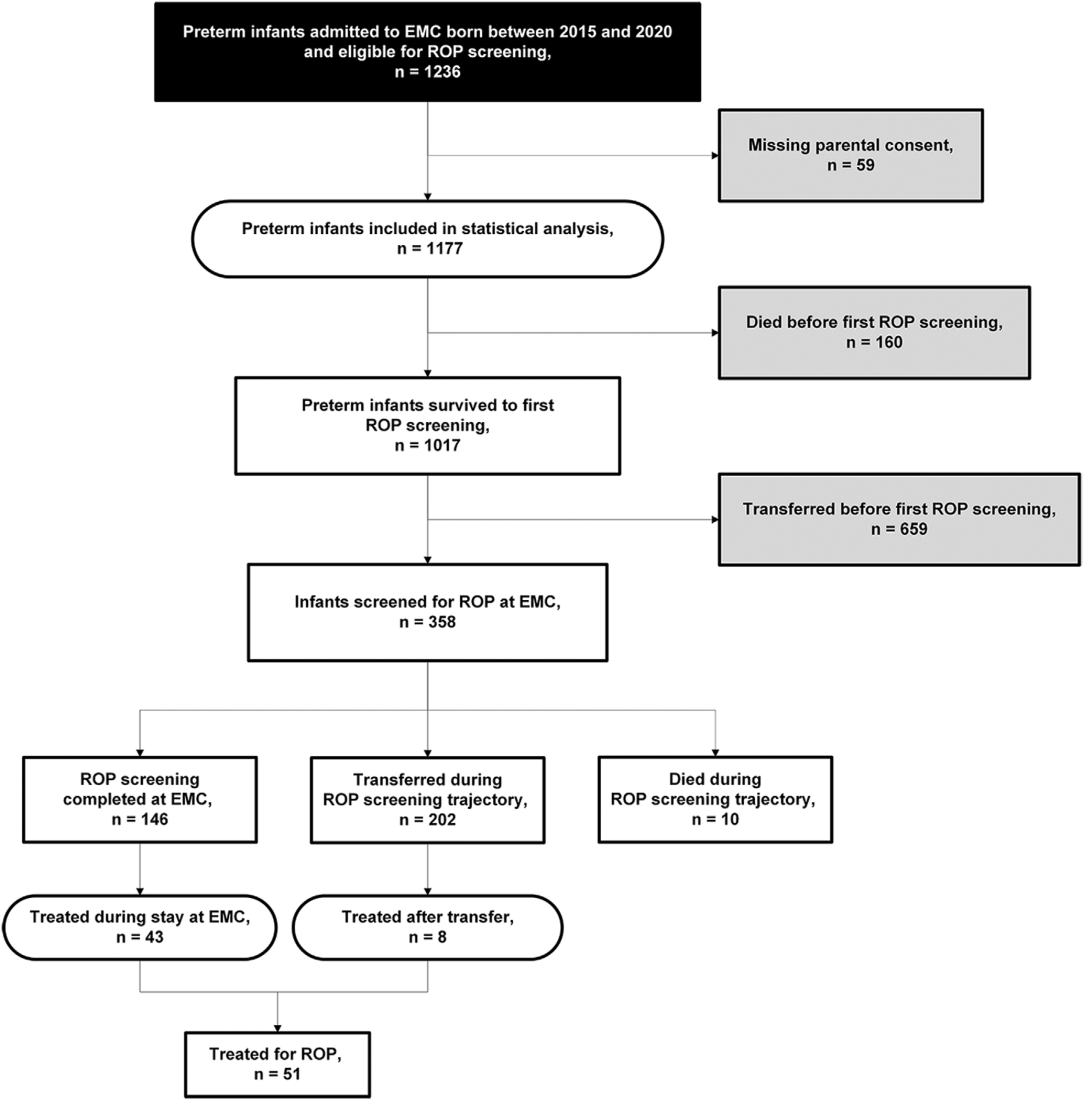
Flowchart of patient inclusion. Flowchart shows the number of infants eligible for ROP screening at Erasmus University Medical Centre Rotterdam (EMC) between 2015 and 2020. Infants are further subdivided based on survival to first screening, screening performed at EMC, screening completion and ROP treatment. EMC, Erasmus University Medical Centre Rotterdam; ROP, retinopathy of prematurity.

A total of 1236 infants met the eligibility criteria for ROP screening. Parents of 59 infants did not consent to the use of their child's health data, resulting in 1177 being included in the statistical analysis (Figure 1). Comparison of demographic characteristics between infants with and without consent revealed no significant differences in gestational age, birthweight, sex, exposure to ROP risk factors or overall mortality (i.e. deaths that occurred before or during the ROP screening trajectory) (Table S3).

The flowchart in Figure 1 shows the further subdivision of eligible infants by survival up until the first ROP screening, actual screening performed at our hospital, screening completion at our hospital and ROP treatment. Of the total cohort, 14% (160/1177) died before the first ROP screening. Of the 1017 surviving infants, 659 (65%) were transferred to other hospitals before ROP screening was set to be initiated, leaving 358 infants (35%) that were screened at our hospital.

Table 1 shows comparisons of demographics based on survival and screening status at our hospital. Infants who died before first screening had lower median gestational age and birthweight, higher rates of mechanical ventilation, NEC and treatment with inotropics, and a higher overall risk summary score, compared with infants who survived. For surviving infants, those who were transferred before first ROP screening had higher median gestational age and birthweight, lower frequencies of all described ROP risk factors, and lower overall risk summary score, compared with those screened at our hospital.

**TABLE 1:** Population demographics of included infants eligible for ROP screening subdivided by survival and screening status.

Characteristics	Total included, (*N* = 1177)	Died before first screening, (*N* = 160)	Survived to first screening, (*N* = 1017)	*p* Value	Transferred before first screening, (*N* = 659)	Screened at EMC, (*N* = 358)	*p* Value
GA (weeks), median (IQR; min–max)	28.6 (3.4; 23.9–35.3)	26.1 (3.0; 23.9–32.7)	28.9 (3.1; 24.0–35.3)	<0.001*	29.6 (2.3; 24.9–35.3)	26.6 (2.4; 24.0–33.9)	<0.001*
BW (grams), median (IQR; min–max)	1050.0 (465.0; 360–3000)	800.0 (362.8; 360–2380)	1100.0 (440.0; 410–3000)	<0.001*	1200.0 (355.0; 685–3000)	857.5 (328.8; 410–2230)	<0.001*
Female, *n* (%)	508 (43)	64 (40)	444 (44)	0.385	299 (45)	145 (41)	0.134
ROP risk factors							
Missing	2	0	2	N/A	2	0	NA
Sepsis, *n* (%)^a^	337 (29)	53 (33)	284 (28)	0.181	103 (16)	181 (51)	<0.001*
Mechanical ventilation, *n* (%)^a^	706 (60)	149 (93)	557 (55)	<0.001*	258 (39)	299 (84)	<0.001*
NEC, *n* (%)^a^	126 (11)	51 (32)	75 (7)	<0.001*	17 (3)	58 (16)	<0.001*
Inotropics, *n* (%)^a^	236 (20)	113 (71)	123 (12)	<0.001*	26 (4)	97 (27)	<0.001*
Steroids, *n* (%)^a^	143 (12)	24 (15)	119 (12)	0.239	4 (0.6)	115 (32)	<0.001*
Risk summary score^b^, median (IQR)^a^	1 (2)	2 (1)	1 (2)	<0.001*	0 (1)	2 (2)	<0.001*

*Note*: Differences between the groups were assessed using Mann–Whitney *U*‐test for numerical variables and Pearson's chi‐squared test for categorical variables.

Abbreviations: BW, birthweight; EMC, Erasmus University Medical Centre Rotterdam; GA, gestational age; IQR, interquartile range; NEC, necrotising enterocolitis; ROP, retinopathy of prematurity.

^a^ % or median of the infants with complete data availability.

^b^ Cumulative number of risk factors per individual infant.

* Statistically significant difference with Bonferroni correction for nine tests *p* < 0.0055.

Table S4 presents gestational age, birthweight and incidence of ROP risk factors in the included cohort in comparison with incidences found in Dutch population‐based studies (Heida et al., 2017; Trzcionkowska, Vehmeijer, et al., 2021).

### 3.2 ROP treatment rate

Of the 358 infants screened at our hospital during the study period, data were complete for 343 infants (96%). Incomplete data (*n* = 15) were a result of limited correspondence after transfers to other hospitals after the first ROP screening. With the number of infants with complete data as the denominator, the incidence of ROP was 81% (279/343), and the incidence of ROP treatment was 15% (51/343). Distributions of ROP stage, zone and presence of plus disease that make up the maximum ROP diagnoses are shown in Table 2. The primary treatment was laser therapy in all cases. In two cases, the infant received a perioperative anti‐VEGF injection in one eye in addition to laser because of impaired laser uptake and obstructed visualisation of the retina by the tunica vasculosa lentis.

**TABLE 2:** Distribution of maximum ROP diagnosis of screened infants.

Maximum ROP diagnosis (*n*, %)	Total (*N* = 358)
Stage	
No ROP	64 (18)
ROP 1	111 (31)
ROP 2	69 (19)
ROP 3	49 (14)
ROP 4a	1 (0.3)
ROP 4b	2 (0.6)
ROP 5	3 (0.8)
Missing^a^	59 (17)
Zone	
No ROP	64 (18)
Zone 3	37 (10)
Zone 2	179 (50)
Zone 1	10 (3)
Missing^a^	68 (19)
Plus disease	
No ROP	64 (18)
No plus	163 (46)
Pre‐plus	26 (7)
Plus	40 (11)
Missing^a^	65 (18)
ETROP type 1	38 (11)
ETROP type 2	25 (7)
ROP stage 3 or higher	55 (15)

Abbreviations: ETROP, Early Treatment of ROP Study; ROP, retinopathy of prematurity.

^a^ Missing cases include infants without a discharge letter available describing their hospital stay after they were transferred, meaning that it was unknown if maximum ROP diagnosis was reached at Erasmus University Medical Centre Rotterdam or after transfer, or a discharge letter is available, but ROP diagnosis is only partly described.

Aside from 358 infants screened at our hospital, another 659 infants survived to first ROP screening, but were already transferred to other hospitals before screening could be initiated. When combining both groups of surviving infants, data could be completed for 70% (716/1017) of cases. Three additional treated cases were identified among the early transfers. With the addition of infants that were transferred to other hospitals at an early stage, the ROP treatment rate lowered to 8% (54/716).

### 3.3 Outsourcing ROP treatment

Since 2018, infants requiring ROP treatment have been referred from our hospital to another tertiary NICU, that is the ‘ROP referral centre’. Table 3 presents screening and treatment characteristics stratified by time period: prior to outsourcing (Period 1) and after outsourcing was implemented (Period 2). The incidence of any‐stage ROP among screened infants with available data was higher in Period 1 (87%, 144/165) compared with Period 2 (76%, 135/178). There was no significant difference in treatment rates among infants diagnosed with ROP between the two time periods.

**TABLE 3:** ROP screening and treatment characteristics in Period 1 (2015–2017) and Period 2 (2018–2020).

Characteristics	Total	Period 1	Period 2	*p* Value
Number of screened infants, *n*	358	167	191	NA
ROP detection, *n* (%)^a^				
Missing	15	2	13	
No ROP	64 (19)	21 (13)	43 (24)	0.007*
ROP	279 (81)	144 (87)	135 (76)	
ROP treatment, *n* (%)^b^				
Not treated	228 (82)	124 (86)	104 (77)	0.05
Treated	51 (18)	20 (14)	31 (23)	
Type of primary treatment, *n* (%)^c^				
Laser OU	49 (96)	18 (90)	31 (100)	NA
Laser OU + anti‐VEGF OD or OS	2 (4)	2 (10)	0 (0)	
Anti‐VEGF OU	0 (0)	0 (0)	0 (0)	
Primary treatment indication, *n* (%)^d^				
Missing	3	0	3	
ETROP type 1	38 (79)	19 (95)	19 (68)	NA
ETROP type 2	6 (13)	0 (0)	6 (21)	
APROP	1 (2)	1 (5)	0 (0)	
Other	3 (6)	0 (0)	3 (11)	
Primary treatment location, *n* (%)^c^				
On‐site	25 (49)	18 (90)	6 (19)	NA
Outsourced	18 (35)	1 (5)	18 (58)	
Treatment initiated after transfer	8 (16)	1 (5)	7 (23)	
Time interval to treatment (days), mean (SD, min–max)^e^	4.0 (2.2, 0–7)	3.0 (2.1, 0–7)	4.5 (1.9, 1–7)	0.014*
Number of infants receiving retreatment, *n*	7	5	2	NA
Number of retreatments, *n*	10	8	2	NA
Type of retreatment, *n* (%)^f^				
Laser	1 (10)	0 (0)	1 (50)	NA
Anti‐VEGF OD or OS	1 (10)	1 (12)	0 (0)	
Anti‐VEGF OU	2 (20)	2 (25)	0 (0)	
Vitrectomy OD or OS	5 (50)	5 (63)	0 (0)	
Vitrectomy OU	1 (10)	0 (0)	1 (50)	
Number of infants developing retinal detachment, *n*	6	5	1	NA

*Note*: Period 1 and Period 2 were compared using the Pearson chi‐squared test (categorical) or independent sample *t*‐test (numerical).

Abbreviations: APROP, aggressive posterior ROP; OD, oculus dexter; OS, oculus sinister; OU, oculus uterque; ROP, retinopathy of prematurity; VEGF, vascular endothelial growth factor.

^a^ % of the number of screened infants complete data availability.

^b^ % of the number of infants diagnosed with ROP.

^c^ % of the number of treated infants.

^d^ % of the number of treated infants with complete data availability.

^e^ Number of days between the decision to treat and actual treatment taking place, there were four missing values for Period 2 (2018–2020).

^f^ % of the number of retreatments.

* Statistically significant difference with Bonferroni correction for three tests *p* < 0.0166.

Although the policy had not yet shifted to outsourcing ROP treatment, two infants were referred to the ROP referral centre during Period 1. One was described to have ‘aggressive posterior ROP’ (APROP), currently called aggressive ROP (AROP), and was treated by an experienced surgeon at the ROP referral centre. The second infant developed treatment‐requiring ROP after transfer from our tertiary referral NICU to a lower‐level care unit at a regional hospital, and was likewise treated at the ROP referral centre. Conversely, six infants were not referred to the ROP referral centre during Period 2, even though the policy had changed to outsourcing treatment. In all six cases, the infants were too clinically unstable to travel, and therefore, one of the ROP referral centre's surgeons travelled to our hospital with laser equipment to perform the procedure on‐site. In addition, seven infants treated in Period 2 developed treatment‐requiring ROP after transfer to a regional hospital: six were also treated at the ROP referral centre, one was treated at another tertiary NICU.

Regarding the specific indications for ROP treatment, there were three cases where the exact ROP diagnosis at treatment could not be retrieved. These were all cases where the infant developed treatment‐requiring ROP after transfer to a regional hospital's level I or II NICU in Period 2. Table 3 shows that 39 out of 48 treated infants with available data were treated for ROP that was classified as ETROP type 1 ROP or aggressive posterior ROP (APROP). Nine infants received treatment for milder forms of ROP, of which six treatments were for ETROP type 2 ROP and three treatments were for non‐threshold disease. Treated cases of non‐threshold disease were all cases of ROP 2 zone 2 with pre‐plus disease. When comparing the available treatment indications before and after treatment was outsourced, 32% (9/28) of treated cases in Period 2 had a diagnosis milder than ETROP type 1 ROP, compared with none of the treated cases in Period 1.

Time interval to treatment, defined as the number of days between the decision to treat and actual treatment taking place, was available for 47 of 51 treated infants. For the other four infants, all treated in Period 2, interval data could not be retrieved after transfer to a regional hospital's level I or II NICU. Overall, the mean time interval to treatment was 4.0 days (SD 2.2), with values ranging from 0 to 7 days. Of treated infants with available interval data, 55% (26/47) experienced a delay of more than 3 days between treatment decision and actual treatment. The mean time interval to treatment was significantly longer in Period 2 compared with Period 1 (Table 3), with a mean difference of 1.5 days (95% CI 0.3–2.8 days).

Of the total of 51 treated infants, seven required a total of 10 additional treatments. Six of these seven infants developed a retinal detachment, including one infant with bilateral retinal detachments. In Period 1, 25% (5/20) of treated cases required additional treatment, compared with 6% (2/31) in Period 2. The majority of additional treatments (8 out of 10) and retinal detachments (5 out of 6) occurred in Period 1 (Table 3).

In the as‐treated analysis, 33% (9/27) of referred treatments had a diagnosis milder than ETROP type 1 ROP, compared with 4% (1/24) of the on‐site treatments. The mean time interval to treatment was significantly longer for referred treatment compared with on‐site treatment, with a mean difference of 1.7 days (95% CI 0.5–2.8 days). For the referred treatments, 17% (4/24) of cases required additional treatment, compared with 11% (3/27) in Period 2. The majority of additional treatments (7 out of 10) and retinal detachments (4 out of 6) occurred after on‐site treatment.

## 4 DISCUSSION

This retrospective observational cohort study reports on the ROP treatment rate and the effects of outsourcing laser treatment at a tertiary referral NICU in the Netherlands. During the study period, approximately one in seven infants screened for ROP at our hospital received laser treatment. Although outsourcing ROP treatment to an external facility led to longer delays in ROP treatment, fewer retinal detachments occurred after its implementation.

### 4.1 ROP treatment rate

ROP treatment was administered to 15% of the infants screened at our hospital: a rate substantially higher than previously published national incidences, both in the Netherlands and in other European countries. Nationwide inventories across Europe report treatment rates among screened infants ranging from 1% to 9% (Modrzejewska & Bosy‐Gąsior, 2023), with the most recent Dutch inventory finding an incidence of 3.6% (39/1085) (Trzcionkowska et al., 2023). The high treatment rate found in our cohort is mirrored by similarly disproportionate incidence of any‐stage ROP. The NEDROP 2 study reported that any‐stage ROP was found in 28% (305/1085) of infants screened for ROP in 2017 in the whole of the Netherlands (Trzcionkowska et al., 2023); in contrast, in our cohort, over 80% of all infants screened was diagnosed with ROP. Other national population‐based studies show ROP incidence rates ranging from 12% to 36% (Hong et al., 2022).

The disparity between the national incidences of ROP and ROP treatment and rates observed in our hospital's screened cohort could be explained by the early transfer of low‐risk infants to a regional level I or II NICU, resulting in a concentration of high‐risk infants at our level IV NICU. Almost two‐thirds of all admissions eligible for ROP screening were transferred before the first ROP screening could be performed. Compared with transferred infants of our cohort and compared with national inventory study populations (Heida et al., 2017; Trzcionkowska, Groenendaal, et al., 2021), infants screened at our hospital were smaller, more premature and had higher exposure to ROP risk factors (Table 2 and Table S4), which predisposes these infants to more severe presentations ROP with a greater likelihood of requiring treatment. These findings reflect the regionalised structure of neonatal care in the Netherlands, where clinically stable infants are transferred to a level I or II NICU in regional hospitals closer to home as early as possible.

Given the influence of the concentration of high‐risk infants in our tertiary NICU, comparison with other tertiary or academic centres may be more relevant. However, while centre‐specific treatment rates do occasionally exceed nationwide incidence rates, there are no recent publications reporting treatment in more than 13% of screened cases (Modrzejewska & Bosy‐Gąsior, 2023). In addition, when mitigating the effect of early transfers of low‐risk infants by calculating the treatment rate among all surviving infants, we still find a considerably higher rate than the national incidence rate (3.6%), namely 5% (51/1017), which might even be an underestimation due to missing data. Therefore, additional contributing factors beyond the composition of our cohort should be considered. One such factor may be the considerable number of cases of ROP milder than type 1 ROP that were treated, especially since ROP treatment was outsourced to an external facility. In 19% of treated cases, the infant had not yet developed type 1 ROP (Table 3), and therefore could theoretically have been managed with a watchful waiting approach rather than be treated. Still, these infants could have progressed to type 1 ROP later on, which is why the impact of these cases of possible ‘over‐treatment’ on the overall treatment rate at our hospital is difficult to determine.

### 4.2 Outsourcing ROP treatment

When comparing ROP treatment characteristics before (Period 1, 2015–2017) and after (Period 2, 2018–2020) the implementation of outsourced of ROP treatment, three key differences emerged: (1) the time interval between ROP treatment decision and ROP treatment was significantly longer in Period 2; (2) treatment of ROP milder than type 1 ROP occurred more frequently in Period 2 and (3) less retinal detachments were reported in Period 2.

Regarding the first finding, increased time between treatment decision and actual treatment may be attributed to the logistical challenges that come with planning and executing neonatal transport. Various national ROP guidelines advise treating type 1 ROP within 24–72 h to minimise the risk of retinal detachment, particularly in cases of aggressive ROP (Centre de Référence des Maladies Rares en Ophtalmologie (OPHTARA) Hôpital Universitaire Necker Enfants Malades, 2023; Nederlands Oogheelkundig Gezelschap, 2013; Royal College of Ophthalmologists, 2022; SWEDROP Styrgruppen, 2023; Türk Neonatoloji Derneği, 2021). However, even prior to the introduction of outsourced ROP treatment, the mean interval between treatment decision and actual treatment was already 3 days (Table 3), and delays of more than 3 days were common. This suggests that outsourcing ROP treatment only adds to the already existing factors that impact the timing of treatment, such as limitations in scheduling and capacity issues, even in the absence of inter‐hospital transfer. Given the small number of infants requiring ROP treatment, and the organisational changes made during the study period, it is difficult to assess the impact of delays (>3 days) on the success of primary treatment.

With regard to treatment indications, this study found that treatment of ROP milder than type 1 ROP emerged since outsourced treatment was implemented. A possible explanation is that, due to the organisational change, our hospital's ophthalmologists have anticipated treatment delays by referring infants at an earlier ROP stage. Similar observations have been reported in other studies, were logistical barriers have led to treatment of milder forms of ROP (Gupta et al., 2016; Lemaître et al., 2021). Thus, an unintended consequence of outsourcing ROP treatment might be over‐treatment of ROP, unnecessarily increasing the risk of laser‐associated complications such as refraction errors and visual field defects later in life (Wright et al., 2006). However, previously described telemedicine‐based early referral programmes report transferring infants with referral‐warranted ROP who, if the disease does not progress, ultimately return without treatment (Weaver & Murdock, 2012). Therefore, improving inter‐expert collaboration, particularly if treatment is centralised, may support ophthalmologists in balancing timely intervention with the risk of over‐treatment.

Regarding adverse outcomes, more retinal detachments were reported prior to the transition to outsourcing treatment to the ROP referral centre, that is before 2018. Previous studies from Sweden have indicated that suboptimal laser treatment is a prevalent cause for the need for additional treatment (Spandau et al., 2020), and have highlighted variations in rates for additional treatments across different hospitals (Holmström et al., 2024), and between individual surgeons (Holmström et al., 2016). While the decision to outsource ROP treatment at our hospital was not primarily driven by the aim of enhancing laser outcomes, and despite the relatively small overall number of additional treatments we observed, our results may suggest that directing cases of treatment‐requiring ROP to a more specialised group of surgeons could lead to improved primary treatment success. It is important to note, however, that factors beyond surgeon expertise, such as the use of different laser equipment (diode laser 810 nm or green argon laser 532 nm) and more frequent treatment of ROP milder than type 1 ROP in Period 2, may have also played a role in these findings. Moreover, Period 2 still saw an instance of retinal detachment following primary laser therapy.

Lastly, the as‐treated analysis did not reveal any unexpected findings compared to the time period–based analysis. When combining all infants referred for treatment, delays between treatment decision and intervention were longer than for on‐site treatments, and a higher proportion of referred treatments were performed before meeting criteria for type 1 ROP. Although the majority of retinal detachments (four out of six) occurred following on‐site treatment, the absolute distribution was slightly more balanced than in the time period–based analysis (five out of six), due to the nature of the referral process in Period 1 (i.e. referral of a case of aggressive ROP that resulted in retinal detachment).

There are limitations to this study. First, to explain the high incidences for ROP and ROP treatment at our hospital, only the ROP risk factors incorporated in the national ROP screening guidelines were studied, as those are deemed most relevant to the Dutch population of preterm infants at risk for ROP (Trzcionkowska, Vehmeijer, et al., 2021; van Sorge et al., 2013, 2014). Although this falls outside the scope this study, incorporating other prenatal or antenatal risk factors, biomarkers or genetic indicators (Almutairi et al., 2024; El Emrani et al., 2024; Holmström et al., 2007; Kim et al., 2018) might further explain the high overall occurrence of ROP at our hospital. Second, ROP screening via retinal camera was introduced in 2017. This change in screening modality might have impacted peer consultation and patient handover, for example, to external experts. Third, the missing values for ROP type at treatment decision and time interval to treatment, although small in number, were concentrated within the referred treatment group due to the higher number of cases where treatment was initiated after transfer to a regional hospital's level I or II NICU. While the same logistical issues will have probably affected treatment initiated elsewhere, the lack of complete information might have swayed results. Lastly, the medical transport necessary for ROP treatment at an external facility may impact the child's clinical condition and may lead to additional healthcare costs. These factors have not been addressed in this research.

In conclusion, the incidence of ROP at our tertiary referral NICU exceeds nationally reported rates, and a comparatively high proportion of infants undergo treatment. Our findings provide ophthalmologists and neonatologists better insight into how transfer patterns and treatment considerations affect hospital‐level incidences of ROP and ROP treatment, which could help anticipate demand for ROP‐related care at our hospital. ROP screening and treatment are integral parts of neonatal intensive care, and well‐organised ROP screening and treatment are essential to safeguarding future visual function of preterm infants. Our results suggest that outsourced ROP treatment could be a good alternative to on‐site ROP treatment when faced with a shortage of specialised staff, and might even lead to improved treatment success, that is less retinal detachments. Nevertheless, outsourced treatment can come with unforeseen logistical challenges, which could potentially result in treatment delays or over‐treatment. Future research should further explore the full scope of the advantages and disadvantages of centralising ROP treatment.

## AUTHOR CONTRIBUTIONS

L. A. Derks, S. E. Loudon, J. R. Vingerling and A. M. Tjiam contributed to the conceptualisation and design of this study. L. A. Derks acquired the data. L. A. Derks, S. E. Loudon, H. R. Taal, I. K. M. Reiss, J. R. Vingerling and A. M. Tjiam contributed to the data analysis and interpretation of findings. L. A. Derks, S. E. Loudon, H. R. Taal, I. K. M. Reiss, J. R. Vingerling and A. M. Tjiam critically reviewed and approved the final manuscript for final publication.

## FUNDING INFORMATION

LD has received funding from Landelijke Stichting voor Blinden & Slechtzienden (LSBS), Rotterdamse Stichting Blindenbelangen (RSB), For Wis(h)dom Foundation, Stichting Per Pugnam Quod Ames (PPQA) and Prof. Dr. Henkes Stichting. None of these organisations were involved in the design or conduct of this study.

## Supporting information


Table S1

Table S2

Table S3

Table S4

## Data Availability

All data generated during this study are presented in this manuscript and supporting information files. The data that support the findings of this study are available on request from the corresponding author. The data are not publicly available due to privacy or ethical restrictions.

## References

[aos70029-bib-0001] Almutairi, M. , Chechalk, K. , Deane, E. , Fox, R. , Janes, A. , Maguire‐Henry, T. et al. (2024) Biomarkers in retinopathy of prematurity: a systematic review and meta‐analysis. Frontiers in Pediatrics, 12, 1371776. 38571701 10.3389/fped.2024.1371776 PMC10987861

[aos70029-bib-0002] Blencowe, H. , Lawn, J.E. , Vazquez, T. , Fielder, A. & Gilbert, C. (2013) Preterm‐associated visual impairment and estimates of retinopathy of prematurity at regional and global levels for 2010. Pediatric Research, 74 Suppl 1, 35–49. 24366462 10.1038/pr.2013.205 PMC3873709

[aos70029-bib-0003] Centre de Référence des Maladies Rares en Ophtalmologie (OPHTARA) Hôpital Universitaire Necker Enfants Malades . (2023) Protocole National de Diagnostic et de Soins (PNDS): Prise en charge de la Rétinopathie du prématuré.

[aos70029-bib-0004] Davenport, M. , De Ville de Goyet, J. , Stringer, M.D. , Mieli‐Vergani, G. , Kelly, D.A. , McClean, P. et al. (2004) Seamless management of biliary atresia in England and Wales (1999–2002). Lancet, 363, 1354–1357. 15110492 10.1016/S0140-6736(04)16045-5

[aos70029-bib-0005] Davenport, M. , Ong, E. , Sharif, K. , Alizai, N. , McClean, P. , Hadzic, N. et al. (2011) Biliary atresia in England and Wales: results of centralization and new benchmark. Journal of Pediatric Surgery, 46, 1689–1694. 21929975 10.1016/j.jpedsurg.2011.04.013

[aos70029-bib-0006] El Emrani, S. , Jansen, E.S.J. , Goeman, J.J. , Termote, J.U.M. , Lopriore, E. , Schalij‐Delfos, N.E. et al. (2024) Enhancing the retinopathy of prematurity risk profile through placental evaluation of maternal and fetal vascular Malperfusion. Investigative Ophthalmology & Visual Science, 65, 9. 10.1167/iovs.65.11.9 PMC11379085 39230991

[aos70029-bib-0007] Gilbert, C. (2008) Retinopathy of prematurity: a global perspective of the epidemics, population of babies at risk and implications for control. Early Human Development, 84, 77–82. 18234457 10.1016/j.earlhumdev.2007.11.009

[aos70029-bib-0008] Gilbert, C. , Malik, A.N.J. , Nahar, N. , Das, S.K. , Visser, L. , Sitati, S. et al. (2019) Epidemiology of ROP update – Africa is the new frontier. Seminars in Perinatology, 43, 317–322. 31151778 10.1053/j.semperi.2019.05.002

[aos70029-bib-0009] Good WV & Early Treatment for Retinopathy of Prematurity Cooperative Group . (2004) Final results of the Early Treatment for Retinopathy of Prematurity (ETROP) randomized trial. Transactions of the American Ophthalmological Society, 102, 233–248; discussion 248–250. 15747762 PMC1280104

[aos70029-bib-0010] Gupta, M.P. , Chan, R.V.P. , Anzures, R. , Ostmo, S. , Jonas, K. , Chiang, M.F. et al. (2016) Practice patterns in retinopathy of prematurity treatment for disease milder than recommended by guidelines. American Journal of Ophthalmology, 163, 1–10. 26705094 10.1016/j.ajo.2015.12.005 PMC4769781

[aos70029-bib-0011] Heida, F.H. , Stolwijk, L. , Loos, M.H.J. , van den Ende, S.J. , Onland, W. , van den Dungen, F.A.M. et al. (2017) Increased incidence of necrotizing enterocolitis in The Netherlands after implementation of the new Dutch guideline for active treatment in extremely preterm infants: results from three academic referral centers. Journal of Pediatric Surgery, 52, 273–276. 27923478 10.1016/j.jpedsurg.2016.11.024

[aos70029-bib-0012] Hellström, A. , Smith, L.E.H. & Dammann, O. (2013) Retinopathy of prematurity. Lancet, 382, 1445–1457. 23782686 10.1016/S0140-6736(13)60178-6 PMC4389630

[aos70029-bib-0013] Holmström, G. , Hellström, A. , Jakobsson, P. , Lundgren, P. , Tornqvist, K. & Wallin, A. (2016) Five years of treatment for retinopathy of prematurity in Sweden: results from SWEDROP, a national quality register. The British Journal of Ophthalmology, 100, 1656–1661. 26969711 10.1136/bjophthalmol-2015-307263

[aos70029-bib-0014] Holmström, G. , Hellström, A. , Teär Fahnehjelm, K. , Gränse, L. , Sandgren Hochhard, K. , Sunnqvist, B. et al. (2024) Treatment for retinopathy of prematurity in Sweden 2008‐2021: reduced gestational age of treated infants and remaining differences in treatment type and recurrence rates between hospitals. Acta Ophthalmologica, 102, 401–408. 37698061 10.1111/aos.15751

[aos70029-bib-0015] Holmström, G. , Van Wijngaarden, P. , Coster, D.J. & Williams, K.A. (2007) Genetic susceptibility to retinopathy of prematurity: the evidence from clinical and experimental animal studies. The British Journal of Ophthalmology, 91, 1704–1708. 18024814 10.1136/bjo.2007.117283 PMC2095497

[aos70029-bib-0016] Hong, E.H. , Shin, Y.U. & Cho, H. (2022) Retinopathy of prematurity: a review of epidemiology and current treatment strategies. Clinical and Experimental Pediatrics, 65, 115–126. 34645255 10.3345/cep.2021.00773 PMC8898617

[aos70029-bib-0017] International Committee for the Classification of Retinopathy of Prematurity . (2005) The international classification of retinopathy of prematurity revisited. Archives of Ophthalmology, 123, 991–999. 16009843 10.1001/archopht.123.7.991

[aos70029-bib-0018] Kim, S.J. , Port, A.D. , Swan, R. , Campbell, J.P. , Chan, R.V.P. & Chiang, M.F. (2018) Retinopathy of prematurity: a review of risk factors and their clinical significance. Survey of Ophthalmology, 63, 618–637. 29679617 10.1016/j.survophthal.2018.04.002 PMC6089661

[aos70029-bib-0019] Lemaître, D. , Barjol, A. , Abdelmassih, Y. , Farnoux, C. , Martin, G.C. , Metge, F. et al. (2021) Treatment outside the recommended guidelines for retinopathy of prematurity (ROP): prevalence, characteristics, and issues. Journal of Clinical Medicine, 11, 39. 35011779 10.3390/jcm11010039 PMC8745039

[aos70029-bib-0020] Leoni, J. , Rougemont, A.L. , Calinescu, A.M. , Ansari, M. , Compagnon, P. , Wilde, J.C.H. et al. (2022) Effect of centralization on surgical outcome of children operated for liver tumors in Switzerland: a retrospective comparative study. Children (Basel), 9, 217. 35204937 10.3390/children9020217 PMC8870146

[aos70029-bib-0021] Modrzejewska, M. & Bosy‐Gąsior, W. (2023) Most up‐to‐date analysis of epidemiological data on the screening guidelines and incidence of retinopathy of prematurity in Europe‐a literature review. Journal of Clinical Medicine, 12, 3650. 37297844 10.3390/jcm12113650 PMC10253523

[aos70029-bib-0022] Nederlands Oogheelkundig Gezelschap . (2013) Richtlijn prematuren retinopathie (ROP).

[aos70029-bib-0023] Perined . (2022) Peristat.

[aos70029-bib-0024] Royal College of Ophthalmologists . (2022) Clinical guidelines – Treating retinopathy of prematurity in the UK.

[aos70029-bib-0025] Söderström, L. , Graneli, C. , Rossi, D. , Hagelsteen, K. , Gunnarsdottir, A. , Oddsberg, J. et al. (2024) National centralization of Hirschsprung's disease in Sweden: a comparison of postoperative outcome. Pediatric Surgery International, 40, 265. 39369074 10.1007/s00383-024-05842-6 PMC11455800

[aos70029-bib-0026] Spandau, U. , Larsson, E. & Holmström, G. (2020) Inadequate laser coagulation is an important cause of treatment failure in type 1 retinopathy of prematurity. Acta Ophthalmologica, 98, 795–799. 32250547 10.1111/aos.14406

[aos70029-bib-0027] Stark, A.R. , Pursley, D.M. , Papile, L.A. , Eichenwald, E.C. , Hankins, C.T. , Buck, R.K. et al. (2023) Standards for levels of neonatal care: II, III, and IV. Pediatrics, 151, e2023061957. 37212022 10.1542/peds.2023-061957

[aos70029-bib-0028] SWEDROP Styrgruppen . (2023) Nationella riktlinjer för screening och behandling av prematuritetsretinopati (ROP).

[aos70029-bib-0029] Trzcionkowska, K. , Groenendaal, F. , Andriessen, P. , Dijk, P.H. , van den Dungen, F.A.M. , van Hillegersberg, J.L. et al. (2021) Risk factors for retinopathy of prematurity in The Netherlands: a comparison of two cohorts. Neonatology, 118, 462–469. 34293743 10.1159/000517247

[aos70029-bib-0030] Trzcionkowska, K. , Termote, J.U.M. , Böhringer, S. , van Sorge, A.J. & Schalij‐Delfos, N.E. (2023) Nationwide inventory on retinopathy of prematurity screening in The Netherlands. British Journal of Ophthalmology, 107, 712–716. 34893474 10.1136/bjophthalmol-2021-319929 PMC10176329

[aos70029-bib-0031] Trzcionkowska, K. , Vehmeijer, W.B.H.J. , Kerkhoff, F.T. , Bauer, N.J.C. , Bennebroek, C.A.M. , Dijk, P.H. et al. (2021) Increase in treatment of retinopathy of prematurity in The Netherlands from 2010 to 2017. Acta Ophthalmologica, 99, 97–103. 32701185 10.1111/aos.14501 PMC7891652

[aos70029-bib-0032] Türk Neonatoloji Derneği . (2021) Türkiye Prematüre Retinopatisi Rehberi 2021 Güncellemesi.

[aos70029-bib-0033] van der Steeg, A.F.W. , Jans, M. , Tytgat, G. , Fiocco, M.F. , van de Ven, C. , Terwisscha, C.E.J. et al. (2023) The results of concentration of care: surgical outcomes of neuroblastoma in The Netherlands. European Journal of Surgical Oncology, 49, 505–511. 36307270 10.1016/j.ejso.2022.10.005

[aos70029-bib-0034] van Sorge, A.J. , Schalij‐Delfos, N.E. , Kerkhoff, F.T. , van Rijn, L.J. , van Hillegersberg, J.L. , van Liempt, I.L.A. et al. (2013) Reduction in screening for retinopathy of prematurity through risk factor adjusted inclusion criteria. British Journal of Ophthalmology, 97, 1143–1147. 23823079 10.1136/bjophthalmol-2013-303123

[aos70029-bib-0035] van Sorge, A.J. , Termote, J.U.M. , Kerkhoff, F.T. , van Rijn, L.J. , Simonsz, H.J. , Peer, P.G.M. et al. (2014) Nationwide inventory of risk factors for retinopathy of prematurity in The Netherlands. The Journal of Pediatrics, 164, 494–498.e491. 24360994 10.1016/j.jpeds.2013.11.015

[aos70029-bib-0036] Vonlanthen, R. , Lodge, P. , Barkun, J.S. , Farges, O. , Rogiers, X. , Soreide, K. et al. (2018) Toward a consensus on centralization in surgery. Annals of Surgery, 268, 712–724. 30169394 10.1097/SLA.0000000000002965

[aos70029-bib-0037] Weaver, D.T. & Murdock, T.J. (2012) Telemedicine detection of type 1 ROP in a distant neonatal intensive care unit. Journal of AAPOS, 16, 229–233. 22681938 10.1016/j.jaapos.2012.01.007

[aos70029-bib-0038] Wijnen, M.H.W.A. & Hulscher, J.B.F. (2022) Centralization of pediatric surgical care in The Netherlands: lessons learned. Journal of Pediatric Surgery, 57, 178–181. 34836641 10.1016/j.jpedsurg.2021.10.023

[aos70029-bib-0039] Wright, K.W. , Spiegel, P.H. & Thompson, L.S. (2006) Handbook of pediatric retinal disease. New York, NY: Springer‐Verlag New York Inc.

