# Supplementary material for: Management of retinopathy of prematurity in a tertiary referral neonatal intensive care unit: Treatment rates and the impact of outsourcing laser therapy
Source: Acta Ophthalmol. 2025 Nov 25;104(4):e416–25. doi: 10.1111/aos.70029 (PMC13166394; doi:10.1111/aos.70029)
Supplement: Supplementary file 1 — Table S1 [file AOS-104-e416-s001.pdf]

Table S1: Dutch ROP screening guidelines 2012 – Table provides an overview of the Dutch ROP screening guidelines (1), including eligibility criteria, timing of first and follow-up screening examinations, and criteria for ending screening.

**Eligibility for screening**	All infants with GA <30 weeks and/or BW <1250 grams<br><br>All infants with GA 30-32 weeks and/or BW 1250-1500 grams and one or more of the following risk factors:<br>• artificial ventilation<br>• sepsis<br>• necrotising enterocolitis (NEC)<br>• postnatal use of corticosteroids<br>• use of cardiotonica to combat hypotension<br>Or: all infants GA<32 weeks and/or BW<1500 grams when reporting of these risk factors are unreliable.
**First screening**	The first screening is recommended at 5 weeks (35-42 days) PNA, but not before 31 weeks PMA.
**Follow-up of screening**	Screening at least twice per week in the case of ROP with plus disease
	Weekly screening in de case of:<br>a. Avascular retina in zone I<br>b. ROP 1-2 in zone I without plus disease<br>c. ROP 2-3 in zone II without plus disease<br>d. Regressing ROP in zone I<br>e. Assessment of the fundus not possible
	Screening once every two weeks in the case of:<br>a. Avascular retina in zone II without ROP<br>b. ROP 1 in zone II<br>c. ROP 1-2 in zone III<br>d. Regressing ROP in zone II-III
**Reducing screening frequency**	The screening frequency can be reduced when:<br>- The severity of ROP decreases in multiple consecutive screening visits;<br>- The infant reaches 40 weeks PMA without ROP.
**Ending screening**	Screening ends when one of the following criteria is met:<br>- Complete vascularisation of the retina;<br>- Apparent regression of ROP at 40 weeks PMA without plus disease;<br>- Apparent growth of the retinal vessels over the demarcation line towards the ora serrata;<br>- Apparent regression of ROP with the ridge changing from pink to white.

Abbreviations: BW, birthweight; GA, gestational age; PMA, post-menstrual age; PNA, post-natal age; ROP, retinopathy of prematurity.

(1) Nederlands Oogheelkundig Gezelschap (2013): Richtlijn Prematuren retinopathie (ROP). https://www.nedrop.nl/upload/definitieve-roprichtlijn-juli-2012.pdf (accessed March 3, 2025)
