# Supplementary material for: Management of retinopathy of prematurity in a tertiary referral neonatal intensive care unit: Treatment rates and the impact of outsourcing laser therapy
Source: Acta Ophthalmol. 2025 Nov 25;104(4):e416–25. doi: 10.1111/aos.70029 (PMC13166394; doi:10.1111/aos.70029)
Supplement: Supplementary file 2 — Table S2 [file AOS-104-e416-s002.pdf]

Table S2: Risk factors for retinopathy of prematurity (ROP) – Table provides definitions used in this study for the ROP risk factors that determine screening eligibility in the Dutch ROP guidelines (1): artificial ventilation, nitric oxide, sepsis, necrotising enterocolitis, corticosteroids and inotropic medication.

**Risk factor**	**Definition**
Artificial ventilation	Invasive respiratory support, including conventional mechanical ventilation, high frequency oscillatory ventilation (HFOV), and synchronous positive pressure ventilation (SIPPV).
Nitric oxide	Treatment of respiratory failure with nitric oxide.
Sepsis	Blood culture-proven sepsis.
Necrotising enterocolitis	Necrotising enterocolitis Bell's stage 2 or higher, regardless of surgery.
Corticosteroids	Post-natal use of corticosteroids because of the pulmonary condition of the child
Inotropes	Use of inotropes to combat hypotension

(1) Nederlands Oogheelkundig Gezelschap (2013): Richtlijn Prematuren retinopathie (ROP). https://www.nedrop.nl/upload/definitieve-roprichtlijn-juli-2012.pdf (accessed March 3, 2025)
