# Supplementary material for: Management of retinopathy of prematurity in a tertiary referral neonatal intensive care unit: Treatment rates and the impact of outsourcing laser therapy
Source: Acta Ophthalmol. 2025 Nov 25;104(4):e416–25. doi: 10.1111/aos.70029 (PMC13166394; doi:10.1111/aos.70029)
Supplement: Supplementary file 3 — Table S3 [file AOS-104-e416-s004.pdf]

Table S3: Comparison of included infants and infants without consent – Table shows the comparison of population demographics between infants eligible for ROP screening that were included in this study and infants eligible for ROP screening without parental consent.

Characteristics	Included in statistical analysis, (N = 1177)	Missing parental consent, (N = 59)	p value
GA (weeks), median (IQR; min-max)	28.6 (3.4; 23.9-35.3)	28.1 (3.9; 24.1-34.4)	0.052
BW (grams), median (IQR; min-max)	1050.0 (465.0; 360-3000)	970.0 (430.0; 450-1985)	0.346
Female, n (%)	508 (43)	25 (42)	0.905
ROP risk factors, n (%)			
Missing	2	0	N/A
Sepsis, n (%)†	337 (29)	19 (32)	0.560
Mechanical ventilation, n (%)†	706 (60)	38 (64)	0.508
NEC, n (%)†	126 (11)	9 (15)	0.277
Inotropics, n (%)†	236 (20)	11 (19)	0.787
Steroids, n (%)†	143 (12)	11 (19)	0.142
Overall mortality*, n (%)	169 (14)	7 (12)	0.593

*Overall mortality includes deaths that occurred before or during the ROP screening trajectory. †% of the infants with complete data availability. Statistically significant difference with Bonferroni correction for 9 tests $p < 0.0055$. Differences between the groups were assessed using Mann Whitney U testing for numerical variables and Chi-square testing for categorical variables. Abbreviations: BW, birthweight; GA, gestational age; IQR, interquartile range; NEC, necrotising enterocolitis; ROP, retinopathy of prematurity.
