# Supplementary material for: Management of retinopathy of prematurity in a tertiary referral neonatal intensive care unit: Treatment rates and the impact of outsourcing laser therapy
Source: Acta Ophthalmol. 2025 Nov 25;104(4):e416–25. doi: 10.1111/aos.70029 (PMC13166394; doi:10.1111/aos.70029)
Supplement: Supplementary file 4 — Table S4 [file AOS-104-e416-s003.pdf]

Table S4: Single-centre and population-based demographics – Table provides an overview of median gestational age and birthweight, and incidences of retinopathy of prematurity (ROP) risk factors on the level of a single tertiary centre and on a national level.

Risk factor	Surviving infants EMC* 2015-2020, N = 1177	Screened EMC† 2015-2020, N = 358	Trzcionkowska et al. (1)‡, 2017, N = 933	Heida et al. (2)§, 2011-2013, N = 1602
GA (weeks), median (min-max)	28.6 (23.9-35.3)	26.6 (24.0-33.9)	28.9 (24.0-32.9)	N/A
BW (grams), median (min-max)	1050.0 (360-3000)	857.5 (410-2230)	1150.0 (410-2510)	N/A
Sepsis, n (%)	337 (29)	181 (51)	353 (38)	N/A
Mechanical ventilation, n (%)	706 (60)	299 (84)	436 (47)	N/A
NEC, n (%)	126 (11)	58 (16)	N/A	137 (9)
Inotropics, n (%)	236 (20)	97 (27)	145 (16)	N/A
Steroids, n (%)	143 (12)	115 (32)	100 (11)	N/A

*Included infants eligible for ROP screening that survived to first ROP screening, regardless of transfer status. †Surviving eligible infants that were actually screened at EMC. ‡A population of infants screened for ROP across the Netherlands that were coupled to the national perinatal registry (Perined). §A population of infants born ≤ 32 weeks gestational age from Amsterdam University Medical Centre, VU University Medical Centre and University Medical Centre Groningen. Abbreviations: BW, birthweight; EMC, Erasmus University Medical Centre Rotterdam; GA, gestational age; NEC, necrotising enterocolitis.

(1) Trzcionkowska K et al. Risk Factors for Retinopathy of Prematurity in the Netherlands: A Comparison of Two Cohorts. Neonatology. 2021;118(4):462-469.

(2) Heida FH et al. Increased incidence of necrotizing enterocolitis in the Netherlands after implementation of the new Dutch guideline for active treatment in extremely preterm infants: Results from three academic referral centres. J Pediatr Surg. 2017;52(2):273-276.
